# Reducing Response Time in Motor Imagery Using A Headband and Deep Learning †

**DOI:** 10.3390/s20236730

**Published:** 2020-11-25

**Authors:** Francisco M. Garcia-Moreno, Maria Bermudez-Edo, José Luis Garrido, María José Rodríguez-Fórtiz

**Affiliations:** Department of Software Engineering, Computer Science School, University of Granada, 18014 Granada, Spain; mbe@ugr.es (M.B.-E.); jgarrido@ugr.es (J.L.G.); mjfortiz@ugr.es (M.J.R.-F.)

**Keywords:** neural networks, deep learning, motor imagery, wearable, EEG, BCI, users’ interaction, response time

## Abstract

Electroencephalography (EEG) signals to detect motor imagery have been used to help patients with low mobility. However, the regular brain computer interfaces (BCI) capturing the EEG signals usually require intrusive devices and cables linked to machines. Recently, some commercial low-intrusive BCI headbands have appeared, but with less electrodes than the regular BCIs. Some works have proved the ability of the headbands to detect basic motor imagery. However, all of these works have focused on the accuracy of the detection, using session sizes larger than 10 s, in order to improve the accuracy. These session sizes prevent actuators using the headbands to interact with the user within an adequate response time. In this work, we explore the reduction of time-response in a low-intrusive device with only 4 electrodes using deep learning to detect right/left hand motion imagery. The obtained model is able to lower the detection time while maintaining an acceptable accuracy in the detection. Our findings report an accuracy above 83.8% for response time of 2 s overcoming the related works with both low- and high-intrusive devices. Hence, our low-intrusive and low-cost solution could be used in an interactive system with a reduced response time of 2 s.

## 1. Introduction

Health care professionals are beginning to use wearable devices for patient monitoring and clinical practice. The wearable data can be analyzed with machine learning (ML) algorithms and consequently predict, prevent, or design an intervention plan [1]. Wearables with different types of built-in sensors (mechanical, physiological, biochemical, and bioimpedance) can measure the physical and mental state of the individuals. They can also act as actuators (output devices), allowing users to interact with the environment. For example, some wearables, such as smart glasses and vibration bracelets (sometimes in combination with other static sensors in the environment, e.g. camera, presence, etc.), provide feedback about users’ interactions or guidance during different activities [2,3,4]. Some commercial wearables could replace expensive medical devices under specific circumstances. Wearables lead to new possibilities in research. Their main benefits are that they are not tied to clinical environments. They can rather be used anytime and anywhere, even outside, i.e., in an ecological way. Additionally, they gather big quantities of data in an objective and precise way. Wearables have been used in evaluation and intervention systems in several healthcare areas, such as healthy ageing, to help the elderly living independently, increasing their autonomy and improving their quality of life [5,6]; and impaired persons [2,7,8].

In particular, some sensors record the brain activity through electroencephalography (EEG) signals to make decisions or to control the environment by evaluating the mind state of the user. Different analyses of data from EEG signals can discover patterns or anomalies of the user’s behavior. Even in the absence of movement, a headband can measure thinking activity, such as motor imagery or goal-oriented thinking, which can be used to interact with a computer system [9]. For example, if the user is thinking about pressing a button on a PC screen, the analysis of the EEG signals could detect such intention, and with the help of an application, actually press the button.

**Figure 1:**
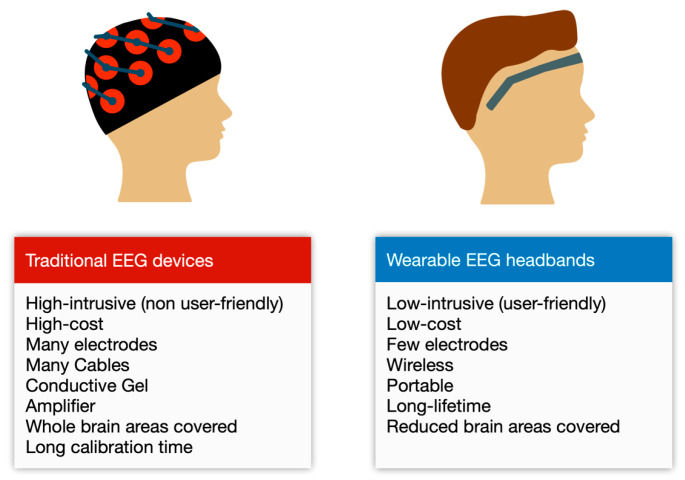
Differences between traditional EEG devices and wearable EEG headbands.

Most of the work performed with EEG signals use intrusive brain computer interfaces (BCI) devices, normally inside hospitals [9]. These traditional BCIs have up to 256 channels (electrodes) and are uncomfortable to wear in outdoor activities, because they need to be in direct contact to the head scalp (it needs even a conductive gel) at different locations, involving expensive machinery and cables paired to the head of the user [10]. The low-cost and low-intrusive EEG headbands, with only a few electrodes, are more comfortable (without cap, cables, and conductive gels) and portable than intrusive EEG devices. Figure 1 summarizes the differences between traditional EEG devices and EEG headbands. EEG headbands could replace traditional EEG machinery for some tasks, especially in combination with ML techniques, which could learn different states of the mind. These headbands prioritize the usability (user-friendly), and the adoption of the headbands by the general public, due to its low-price, versus the accuracy and precision of the data.

Our aim is to explore the potential of low-cost and low-intrusive commercial BCIs on the classification of motor imagery within a short period of time, i.e., that our classifier could detect the motor imagery within a few seconds (less than 10 s) after the subject starts thinking on that movement. In particular, the Muse headband, made by InteraXon [11], of which sensors record electroencephalography (EEG) signals through four channels located around the head of a person. These positions follow the 10–20 international standards [12]. In our previous work [13], we explored the possibilities that the Muse headband offers on the detection of motor imagery. Specifically, we implemented a deep learning (DL) model that could detect left and right hand motor imagery with an accuracy of 98.9%, using data from several users and with 20 s session size. These results, although quite accurate, means that it is not possible for an actuator or application to respond before the session ends. For example, if we need to detect the brain activity of a user in order to press a button (left or right bottom) on a screen, the system needs to wait the 20 s of the session and then classify it (left or right) before responding. Most of the previous works on motor imagery use session sizes bigger than 10 s to ensure they capture the activity of the brain. Only few works use session sizes smaller than 10 s but with intrusive BCI devices. This paper aims at extending our previous work by exploring different session sizes, and therefore to check the response time of an actuator. Specifically, our aim is to check what is the smallest response time that still obtains a decent accuracy on detecting motor imagery. To that end, we performed several experiments with different session and window (splits within the sessions) sizes. Additionally, we improved our previous deep learning model: with different preprocessing techniques, such as averaging data from sliding windows and noise removal; and applying some tuning techniques to prevent overfitting, such as early stopping. Afterwards, we validate our findings by classifying the left/right motor imagery in sessions where the subject is continuously changing directions every few seconds.

The remainder of this paper is organized as follows. Section 2 describes the related work concerning BCI devices and ML algorithms applied to motor imagery, paying special attention to session size. Section 3 introduces the Material and Methods, and also presents the deep learning proposal for classifying two motor imagery tasks: left and right hand movement imagery. Section 4 registers and analyzes the results or our experiments. Finally, Section 5, concludes the paper and describes the future work.

## 2. Related Work

In recent years, deep learning (DL)—a subfield of machine learning—is gaining momentum. An enormous amount of papers using DL have been published in different areas [14,15,16,17]. Health is one of such fields [17] and even DL is commonly used to analyze EEG signals [18]; with obvious restrictions on mobility. During the execution of body part movements or their imagination (motor imagery), such as the hands or eyes, some signals originate in our brain, hence, we can use this correlation to detect movement or intention of movements. Brain activities have different frequencies and can be measured using EEG for driving a BCI system, because they can provide high temporal resolution, reflecting brain dynamic changes in milliseconds [10,19]. The signals measured can be stored and analyzed by means of features extraction to classify the movements with which they are related, inferring the intention of the user to move a specific part of the body, for example, the right or left hand. However, most of the research uses accurate and expensive devices, which are quite intrusive, as the users need to stick sensors all over his/her head, connected with cables to expensive machines [18]. Only a few researchers have experimented with low-cost and low-intrusive EEG devices, such as headbands [18]. For example, Bird et al. [20] used the DL technique long short-term memory (LSTM), which is a type of recurrent neural network (RNN), to learn the attentional emotion and sentiment of one user while providing visual stimuli.

As mentioned above, traditional EEG devices are high-cost, invasive (electrodes implanted in the brain), intrusive and require user-training and long calibration time [19,21]. Although there are non-invasive devices (such as EEG caps) which obtain the EEG data, placing electrodes to the scalp with a conductive gel, they are still high-intrusive because they need an awkward cap with cables linked to machines [10]. Thus, they are uncomfortable to wear in outdoors activities for the users. Nonetheless, there are new wearable devices, usually called BCI headbands. They are low-cost, non-invasive, and low-intrusive, which are more comfortable than intrusive EEG caps, because they do not need conductive gels or any cable. One example of these headbands is Muse, that we used in this research. The low-cost and low-intrusive Muse headband has been used to recognize users and a range of activities (reading, listening to music, playing computer games, watching movies, and relaxing) [22], movements (jaw-clenching and head and eye movements) [23], facial expressions [24], motor imagery [13,25,26], etc.

Most of the previous studies on motor imagery classification applied ML/DL algorithms use EEG recordings greater than 10 s. The BCI devices were both low-intrusive and intrusive. Both Zhang et al. [27] and Chen et al. [28] classified 5 intentions (eyes closed, opening-closing both feet, both fists, left fist, and right fist) with the LSTM technique achieving an accuracy close to 98%, but using 120 s-session size from MI-EEG dataset recorded with a high-intrusive EEG cap (Electro-Cap International USA). Rodriguez et al. [25] achieved 80% accuracy applying convolutional neural networks (CNN) and LSTMs layers to learn 4 motion intentions of a single user (hand, foot, mathematical activity, and relaxation state), but the response time was 30 s. Garcia-Moreno et al. [13] also used CNN+LSTM to classify 2 intentions (right and left hands), reaching 98.9% accuracy for 20 s response time. Li et al. [26] used SVM for classifying left and right hands motor imagery. The average accuracy of the eight participants was 95.1% accuracy (the best individual participant’s accuracy was 98.9%), with a response time of 10 s.

Only few studies on motor imagery detection used EEG-recording durations lower than 10 s. However, either they used intrusive EEG devices or caps (such as Easycap, Germany or Electro-Cap International, USA) placed on the scalp (a conductive gel or paste is needed); or they used public datasets recorded with intrusive devices (such as MI EEG [29], Datasets 2A [30] and 2B [31] of BCI Competition IV [32,33,34,35,36,37,38]. For example, the studies [39,40] were one of the first approaches on motor imagery classification, where they applied logistic regression (LR) and reached an accuracy of 90.5% with recordings of 3 s using intrusive EEG devices. In 2011, Bhattacharyya et al. [32] used 9 s-recordings with an intrusive BCI and applied SVM for left and right motor imagery, achieving 82.14% accuracy, although they use unusual validation split (50–50%, instead of 90–10% or 80–20%). Recent studies [33,34,35,36,37,38] used DL techniques such as CNN and LSTM with recordings of 2–5 s (the best results reached an accuracy of 93.9% with recordings of 4 s [36]), using high-intrusive EEG caps and intra-subject protocol. Two of these recent works [34,37] used only a few electrodes; 3 out of the 22 of the original dataset [31]. In particular, the work of Ha and Jeong [37] achieved the best accuracy, which is 78.44%. They also obtained the results by averaging 9-subject’s accuracies (intra-subject). However, both studies used data recorded with high-intrusive EEG devices. Only one of these recent work [38] reduced the response time to 2 s with a 70% accuracy for intra-subject (by averaging 9-subject’s accuracies) and 40% for cross-subject. However, this work also use data from a high-intrusive EEG cap with 22 electrodes.

In conclusion, previous works on motor imagery focus on the accurate detection of the brain activity, rather than on the response time of a potential actuator. Hence, the session sizes are generally big (bigger than 10 s) to ensure that the activity of the brain is accurately captured (see Table 1, sorted by session size in descending order). Only a few works lower the session size to 4 or 5 s but using intrusive EEG devices. Although some of these works use only 3 or 6 channels of the intrusive device, and therefore, they could resemble a low-intrusive device with only few sensors, the accuracy is below 78% for 4 s session size. We will study in this paper how to lower the session size (lower than 4 s, if possible), but maintaining the accuracy above 80%. We chose 80% because most of the related work using session-sizes up to 4 s report an accuracy lower than 80%.

## 3. Materials and Methods

This section describes the experiment protocols that we have followed; the foundations of the algorithms we have used (deep learning); as well as the workflow proposed for our DL pipeline.

### 3.1. Experiment Protocols

We performed two sets of experiments. The first set explores the reduction of session-sizes and window-sizes, keeping a decent accuracy of the motor imagery detection. To that end, we performed several experiments with different window and session sizes for each motor imagery (right and left). The second set validates the minimum response time achieved in the first set. To that end, we performed several experiments in which the participants where continuously changing direction in motor imagery every few seconds.

Since the movement of eyeballs have an impact on brain waves [26], participants in every experiment rotated the eyes in the corresponding direction while imagining to pick up a bottle of water, but avoiding to move the hand, to touch the bottle or to blink the eyes.

#### 3.1.1. Exploring Response Time Reduction

For the first set of the experiment, we recruited four healthy adults between the ages of 33 and 55 (3 females and 1 male).

**Figure 2:**
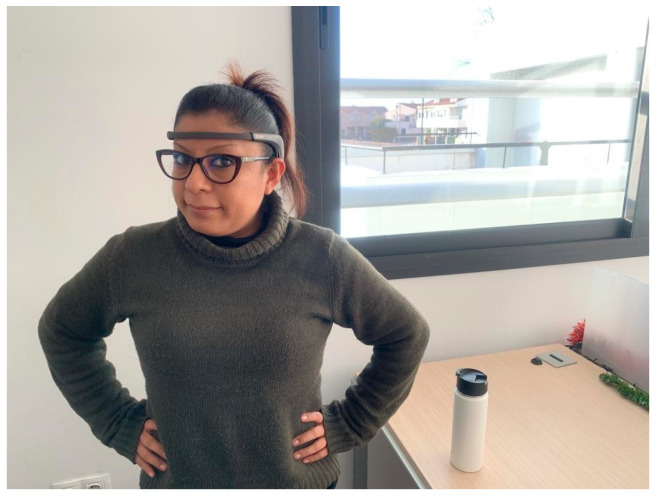
A participant wearing the low-intrusive EEG headband.

We recorded forty EEG sessions per participant with a low-intrusive headband (see Figure 2), performing two different tasks: motor imagery for left and right hands (20 sessions per hand). Each session lasted 20 s and we labelled them (0–1) depending on the tasks (left/right hand). These sessions took place in a silent and distraction-free environment, as the lack of concentration could influence the results.

Summarizing, the experimental protocol consisted of these ordered steps:

1. The participant is sitting down on a chair with arms extended in parallel, resting on a table.
2. A bottle of water is on the table, approximately 5 cm to the left of the left hand.
3. The participant’s head faces forward, while the eyes rotate to the left, looking to the bottle.
4. We asked the participant to imagine picking up the bottle with the left hand, but without moving the hand only thinking about it for 20 s.
5. Then, we asked the participant to relax and to close the eyes for 20 s.
6. We move the bottle on the right side of the table (approximately 5 cm to the right of the right hand).
7. The participant repeats steps 3–5, but for right hand motor imagery.
8. We repeated steps 2–7 for 20 times for each participant.

#### 3.1.2. Validating the Response Time Reduction

To validate the results of the first set of experiments, we created a second set of experiments in which we tried to detect the changes in the motor imagery (right or left) in a continuous session. Due to the restrictions of the pandemic of 2020 and lockdowns, we could not use the same participants of the first experiment. In this second experiment, we recruited three new healthy adults—2 females and 1 male—aging 33–50.

The protocol for this set of experiments consisted of the following ordered steps:

1. The participant is sitting down on a chair with arms extended in parallel, resting on a table.
2. Two bottles of water are on the table. One of them is approximately 5 cm to the left of the left hand and the other bottle is 5 cm to the right of the right hand.
3. The participant’s head face forward, while the eyes rotate to the left, looking to the bottle.
4. We asked the participant to imagine picking up the bottle with the right hand, but without moving the hand; only thinking about it for 6 s.
5. Then, we asked the participant to imagine picking up the bottle with the left hand, but without moving the hand, only thinking about it for 6 s.
6. We repeated steps 4–5 for 5 times for each participant: 1 min in total.
7. Then, we repeated steps 1–6 for 20 times.

### 3.2. Deep Learning Foundations

Deep learning has been traditionally used for computer vision, speech recognition, or natural language processing, and has been extended recently to several fields [15]. Additionally, in EEG signals, DL has been successfully used in the last years [18].

DL applies multiple iterative non-linear transformations of data, simulating the connections of the neurons in a brain. The parameters of the transformations are refined iteratively by minimizing a cost function (i.e., minimizing the error between the predicted and the real signal). DL means several layers of neural networks. However, there is not a consensus on how many layers make it deep. In practice, several DL approaches use just three layers. In a neural network, we have neurons or units organized in these layers: one input layer, one output layer, and one or more hidden layers.

The hidden layers of a deep neural network could be fully connected (FC, also known as the dense layer), recurrent neural network (RNN), or convolutional neural network (CNN). In a FC, all neurons received as input all the weighted outputs of the preceding layer. Generally, it is followed by a non-linear activation function (such as relu and sigmoid). In an RNN, one neuron receives the preceding output of the previous layers and its own output of the previous values.

**Figure 3:**
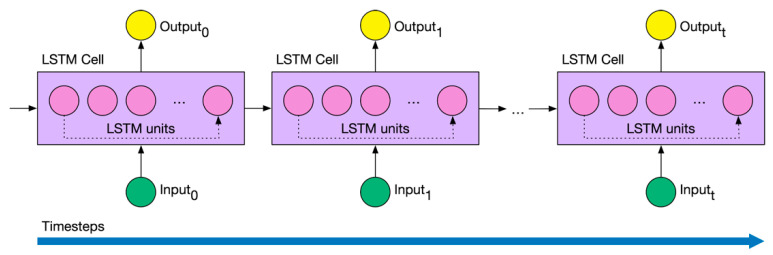
Long short-term memory (LSTM) network diagram.

In particular, one of the most used layers in an RNN is the long short-term memory (LSTM). As LSTM layers are recurrent, they retain memory of the previous time step. This feature makes these layers suitable for time series where the lags between events are uncertain. LSTM hidden layers (neurons) are called units. These units are inside a cell. Like RNN, the LSTM cell has a state and therefore can remember information of the previous timestep (see Figure 3). The common input of an LSTM is a triplet consisting of samples, timesteps, and features.

In a CNN, the outputs are convoluted, and one neuron only takes a subset of output signals (the closest outputs) of the previous layers (depending on the convolution of said outputs). CNN layers detect better the spatial component of the data, which means they can select the best features, and the RNN layers detect better the temporal component of the data [18].

### 3.3. Deep Learning Pipeline

**Figure 4:**
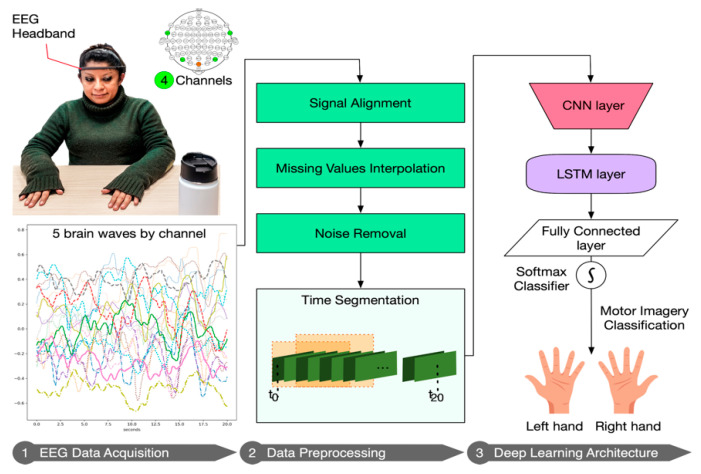
Deep learning pipeline: data acquisition, preprocessing, and deep learning architecture.

This section describes the workflow of our DL pipeline which consists of three main steps: (1) EEG data acquisition; (2) Data preprocessing; and (3) DL architecture for building a model to classify two imagery movement intentions. Figure 4 presents the pipeline which will be explained in detail in the next subsections.

#### 3.3.1. EEG Data Acquisition

**Figure 5:**
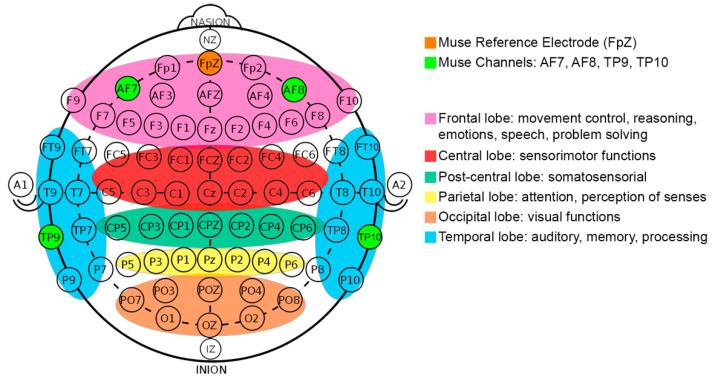
Muse electrodes position and brain functions.

Although previous studies commonly measure hand movements and motor imagery with electrodes located in the primary motor cortex (C3 and C4 electrodes), they use high-intrusive and high-cost EEG devices, which have a lot of cables and electrodes covering all of the lobes of the brain [25,27,28,32,33,34,35,36,39,40] (See Figure 5). Low-cost and low-intrusive EEG headbands have less sensors than the intrusive devices, and do not usually have electrodes in the central area of the brain—motor cortex—(Figure 5, red color). Usually, they have surrounding electrodes located on frontal (Figure 5, pink color) and temporal (Figure 5, blue color) brain areas. The frontal brain lobe is related to brain functions such as movement control, reasoning, emotions, speech, or problem solving [41]. In addition, the temporal lobe is related to auditory stimuli interpretation, meaning, memory, and processing [41]. Thus, the electrodes of these wearables could potentially measure motor imagery. In fact, previous studies [13,25,26] using low-intrusive headbands reached accurate results detecting motor imagery, although they use recordings longer than 10 s (as we presented in Table 1).

We used a Muse headband (version 2) by InteraXon [5], an EEG wearable device with 4 electrodes (channels) which can detect brain signals in a low-intrusive way. This device records EEG using 4 gold-plated cup bipolar electrodes, which are located on the frontal and temporal brain areas. Figure 5 shows (in light green) the location of the electrodes that follows the 10–20 international standard and the brain areas covered. These active electrodes (or channels) are: TP9 (left ear), TP10 (right ear), AF7 (left forehead), AF8 (right forehead) for average sized heads. The frontal electrodes could be placed in the location of F7 and F8 electrodes for small sized heads as Li et al. [26] mentioned. The reference electrode in Muse is Fpz. It does not capture brain signals, but measures the potential differences (voltage) between the active electrodes and Fpz.

Since Software Development Kits (SDK) by InteraXon were discontinued (preventing software developers or researchers to create their custom applications), we used the Mind Monitor application [18] to record the brain signals in real-time.

**Figure 6:**
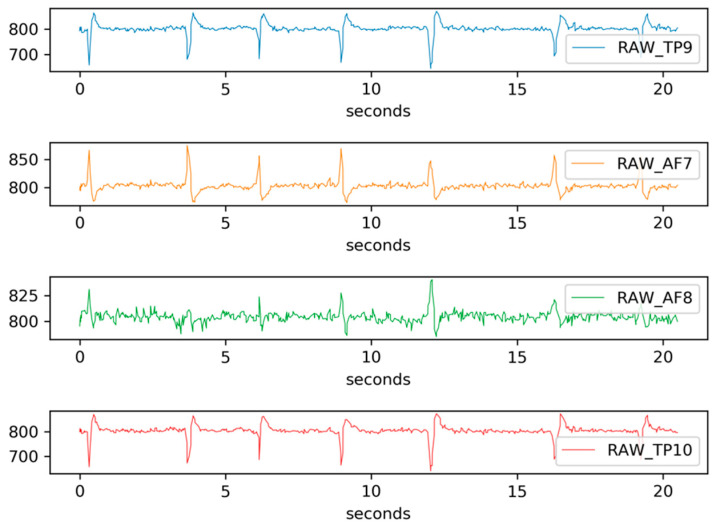
Sample of raw EEG signal data through the four Muse channels.

**Figure 7:**
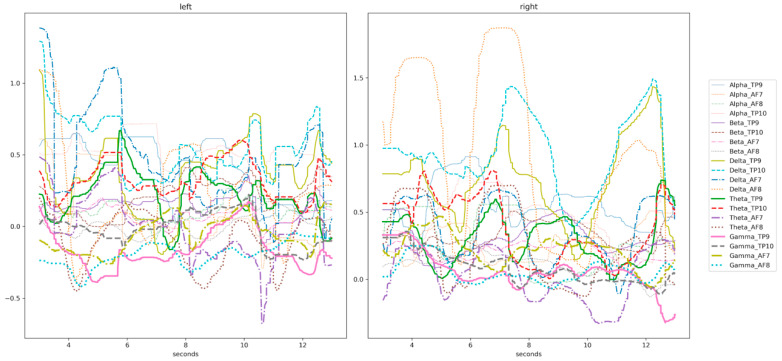
Sample of a participant’s EEG signal for the five waves per channel. The left figure shows the brain waves for left-hand motor imagery and the right figure shows the brain waves for right hand motor imagery.

In the headband, each channel (TP9, TP10, AF7, and AF8) captures a raw EEG signal, measured in microvolts (μV). Thus, we have four raw EEG signal data in total. These raw data consist of 4 signals coming from the 4 channels and they are ranging from 0 to 1682 μV approximately (see a signal sample in Figure 6, ranging from 600 to 800 μV). The Mind Monitor app supports collecting the five common brain waves, or frequency bands (α, β, δ, γ, and θ) and applies a notch filter of 50 Hz to delete electrical power interferences. This app automatically processes the raw data coming from each channel to obtain the brain waves, using the logarithm of the power spectral density (PSD). In ascending order, the frequency bands obtained are: (1) delta (<4 Hz, common in continuous-attention tasks), (2) theta (between 4 and 7 Hz, appears in repressing a response or action), (3) alpha (between 8 and 15 Hz, spikes when relaxing or closing eyes), (4) beta (between 16 and 31 Hz, displays active thinking, focus, high alert, or anxiety), and (5) gamma (>32 Hz, reflects cross-modal sensory processing). Figure 7 shows these 5 waves per each of the 4 channels (in total 20 signals) for left and right hand motor imagery. Furthermore, the headband also records other signals such as accelerometer, heart rate, breath, and muscle movement sensors (allowing to record blinking and jaw clenching). Although, some of these sensors are neither supported for developers nor by the Mind Monitor app.

A signal vector can be represented as follows [X^1^_t = 0_, X^2^_t = 0_, X^3^_t = 0_, X^4^_t = 0_], where X is the point of the signal at time 0 and for the channels 1, 2, 3, or 4 (i.e., TP9, TP10, AF7, and AF8). Therefore, in this way, we can represent the vectors of every wave type replacing “X” for the corresponding Greek letter (α, β, δ, γ, and θ).

#### 3.3.2. Data Preprocessing

First, we need to preprocess a total amount of 20 brain wave signals recorded at a sampling rate of 256 Hz (default value). Since the Mind Monitor app could receive these data with a small delay, we have to align the signals. Additionally, sensors are prone to produce missing values. To avoid inconsistencies in the data, we interpolate the signals to fill up the missing values when possible (small chunks of missing values).

Second, we carried out a noise removal using an exploratory data analysis (EDA) in order to investigate the spatial representation. We could visualize all the signals for left and right hand imagery motions and discarded the noisy signals, mainly due to a lack of contact with headband electrodes on the participant’s skin.

Third, we segmented the resulting data in different session sizes. As we mentioned in previous sections, we have data recorded during a session size of 20 s. Since our aim is to decrease this session size in order to reduce the response time of the prediction, we segmented the data in different session sizes. This preprocessing consisted in getting the signal data from the start of the recording and discarded the rest of the session data; simulating different sessions with different session size. In particular, we use sessions from 1 s to 20 s in intervals of 0.5 s (i.e., [1, 1.5, 2, 2.5, ..., 19.5, 20]). We also use small session sizes of [0.01, 0.025, 0.05, 0.1, 0.25, 0.5] seconds. In addition, we discarded the first two seconds of all recordings in order to prevent some noisy values, due to the setup of each experiment; and the subject needs to start the concentration process.

**Figure 8:**
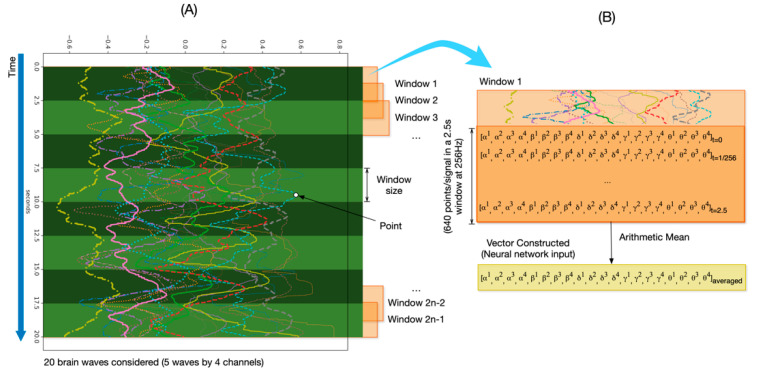
Construction of vector windowed input for a 20-session size applying 50% overlapping of sliding windows. (**A**) Data from one participant with the 20 brain waves (5 waves per each of the 4 channels—AF7, AF8, TP9 and TP10) before segmentation in windows. (**B**) Detailed vector values of a single window and the final vector, calculated as the arithmetic mean of the window vectors.

Fourth, we performed a new data segmentation again (in windows) to each session resulting from the previous segmentation, applying sliding windows with 50% overlapping (see Figure 8). We decided to use overlapping as a data augmentation technique, because this increases the sample size re-using the existing data, contributing to improve accuracy and stability [18]. We extracted the values X^i^_t_, (where i is the channel and t the time) of each signal corresponding to a window size. The number of values per signal depends on the window size and sampling rate (Equation (1)). For example, for a 2.5 s window at a sampling rate of 256 Hz, the number of values of a signal is 640 (“points” in Figure 8). Since the timestep parameter of LSTM layers is the number of windows in which we can split the complete session, we applied the arithmetic mean to average those 640 points of each signal. Hence, we constructed one vector per window. This vector will be the neural network input. For example, if we have a window size of 2 s and sampling rate is 256 Hz, the number of windows (timesteps) will be 512. We tested with several windows sizes (0.01, 0.025, 0.05, 0.1, 0.25, 0.5, 1, 1.5, 2, 2.5, and 5), in order to get the best performance for every session and window (as in previous works [42,43]):

(1) $$\mathrm{signal}\;\mathrm{values}\;\mathrm{per}\;\mathrm{window}\;=\;\frac{\mathrm{window}\;\mathrm{size}\;\left(\mathrm{in}\;\mathrm{seconds}\right)}{\mathrm{sampling}\;\mathrm{rate}\;\left(\mathrm{in}\;\mathrm{seconds}\right)}.$$

Finally, for every window, we can represent the vectors as a concatenation of the 5-wave values, where its components are the values of every wave signal (α, β, δ, γ, and θ) of each EEG channel (In Figure 8, the superindices 1, 2, 3, and 4, corresponding to TP9, TP10, AF7, and AF8). The vectors are represented as:

[α^1^_,_ α^2^_,_ α^3^_,_ α^4^_,_ β^1^_,_ β^2^_,_ β^3^_,_ β^4^_,_ δ^1^_,_ δ^2^_,_ δ^3^_,_ δ^4^_,_ γ^1^_,_ γ^2^_,_ γ^3^_,_ γ^4^_,_ θ^1^_,_ θ^2^_,_ θ^3^_,_ θ^4^]_t = i_ where i is the time, which increases in intervals of 256 Hz (0.00390625 s).

#### 3.3.3. Deep Learning Architecture

**Figure 9:**
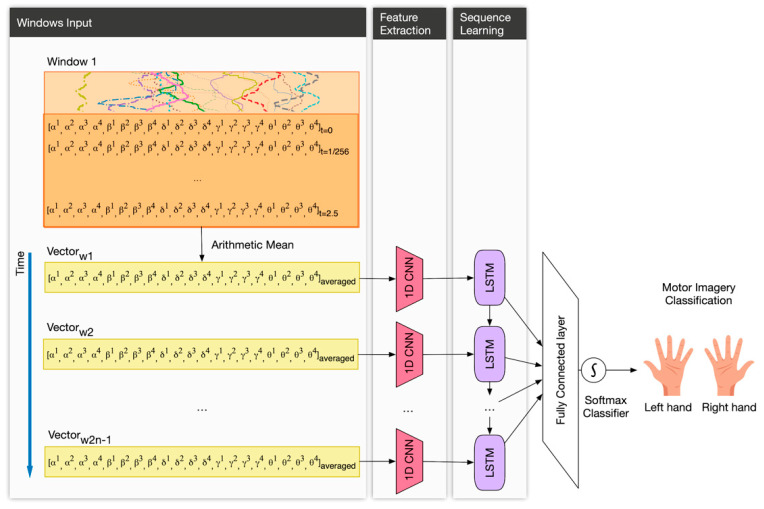
DL Architecture proposed based on 1-dimensional convolutional neural network (1D-CNN) and long short-term memory (LSTM) layers.

In this work, we propose a DL architecture based on 1D-CNN and LSTM layers (Figure 9). The 1D-CNN layer is used to extract the most relevant signals, hereafter referred to as features, (out of the initial 20); and LSTM neural network is used to learn the sequence of the time series. In addition, we tuned the parameters of the layers (e.g., neurons) until we get the best performance. The architecture is the following:

- The input layer. This layer is three-dimensional following this triplet (samples: None, timesteps: number of windows, features: 20). In our case, samples are the number of observations, i.e., the total number of recordings, which the DL model does not know a priori and for that reason is “None”. Since we used 50% overlapping of sliding windows, we defined the timesteps as the number of windows covering the full session (in total, 2n − 1 windows). In addition, features, in our case, are the number of signals, i.e., twenty.
- A 1D-CNN layer with 32 filters of size 1; and kernel size equals 1, which means that each output is calculated based on the previous 1 timestep.
- A LSTM layer with 32 neurons. In order to prevent overfitting, we used 0.1 dropout, 0.0000001 regularizer and early stopping [44]. Early stopping ensures that the neural network stops the training at a certain epoch where further training would overfit the model.
- A fully connected layer with a softmax function activation, for performing the classification of the two motor intentions.

The implementation of the signal processing and the DL model architecture were coded with the Python and Keras library [45]. In Keras, the sample value of the input triplet is commonly set as “None”, because we do not know the total amount of samples to use a priori in the training phase. Therefore, the algorithm will accept any number of samples. Afterwards, when we train the model, we specify the samples split for training and validation.

In the deep learning domain, it is common to split the sample size in three datasets: (1) training; (2) validation: unseen data by the training phase; (3) and testing, unused data in training and validation. Furthermore, for the training and validation, it is common to split the datasets into 80–20% or 90–10% partitions depending on the available dataset size. In our case, we split the data into 80% for building the model and 20% for testing and, finally, we split again this 80% into 80% for training and 20% for validation. Finally, we use accuracy (correct predictions/total predictions) as the performance metric to validate our DL model.

## 4. Results

### 4.1. Set of Experiments 1: Exploring Response Time Reduction

**Figure 10:**
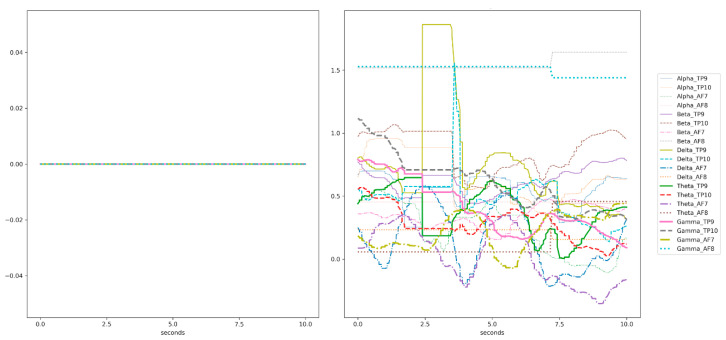
Examples of two noisy signals. (**Left**) Recordings do not show any variation of the signals, possibly due to wrong contact with the skin. (**Right**) Some signals with outliers in Delta TP9/TP10.

Before running the experiments, we discarded several recording sessions (104) that reported a lot of noise in several signals (see Figure 10). For example, Figure 10 in the left-hand size shows that the recordings do not show any variation of the signals. We believe this is due to the wrong contacts of the sensors with the skin of the participant. The right-hand side of Figure 10 shows some signals with outliers. Ultimately, the sample size consisted of 56 samples corresponding to two participants (28 recordings per participant) and with a total recording duration of 17 s (reduced from 20 s because some recordings did not reach 20 s after the preprocessing phase). Although the rest of the participants had some clean recordings, they were discarded because we set a minimum of 14 recordings to compare them, and they do not match this requirement.

**Figure 11:**
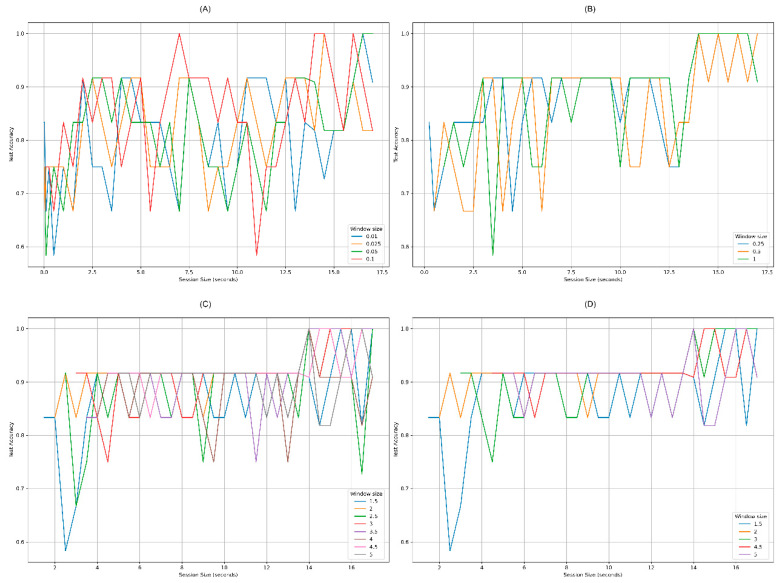
Test accuracy curves for different window sizes. (**A**) Unstable accuracies for very little window sizes. (**B**) Stable accuracies from 6.5 s session size. (**C**) Some stable window sizes from 4 s session size. (**D**) Best stable window sizes between 2 and 4 s of session size.

In Figure 11, we present the results of the experiments. The *x*-axis represents the session size (measured in seconds) and *y*-axis is the test accuracy performance. Each subgraphic corresponds to different window size configurations as we explain below.

As shown in Figure 11 section A, little window sizes (0.01, 0.025, 0.05, 0.1;) reported the worst results, because they never had a stable accuracy over the different session sizes. Intermediate window sizes (0.25, 0.5, and 1; Figure 11 section B) reported good results from 6.5 s of session size onwards. This means that the response time of the prediction has been reduced from 20 s to 6.5. This is already an improvement. Figure 11 section C shows big window sizes (1.5, 2, 2.5, 3, 3.5, 4, 4.5, and 5). These window sizes report also good results from 6.5 s window sessions onwards, varying between 0.75 and 0.9 accuracy for session sizes between 4 and 14 s. Figure 11 section D shows window sizes of 1.5, 2, 3, 4.5, and 5 s that reach an accuracy between 0.83 and 0.92 for session sizes between 2 and 14 s. Summarizing, with small session sizes, we cannot discriminate between left- and right-hands intention; and with big session sizes a user will have to wait for a long response time.

**Figure 12:**
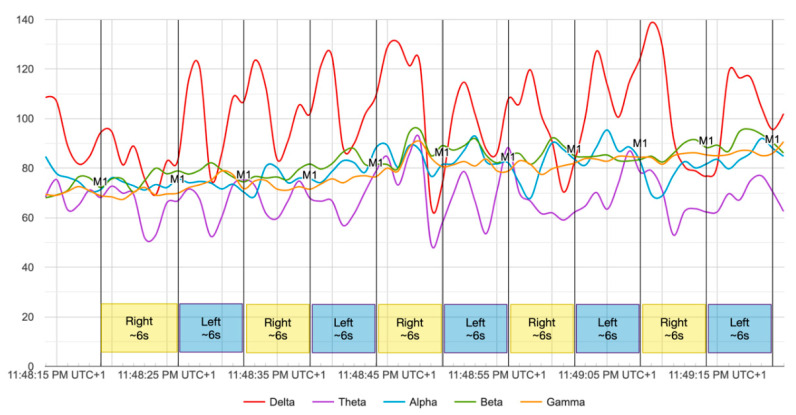
Recording example of experiment 2. M1 is the mark 1, labelled to know when the participant was imaging right/left side. Averaged channel values per wave type.

Therefore, the best stable accuracies appear on windows sizes corresponding to 1.5, 2, 3, 4.5, and 5 s. Since, 1.5 s-window size is stable from 4 s of session size and 2 s-window size is stable from 2 s, we selected 2 s-window size as the best candidate for our DL model, which reduces to 2 s the time-response for the motor imagery classification. In particular, the accuracy of 2 s-window size, ranged from 0.83 and 0.92 approximately for session sizes between 2 and 13.5 s (Figure 12). After 14 s it reaches 0.99 accuracy. This configuration—2 s-window size—maintains a balance between the time-response and an acceptable accuracy performance, because it is able to make an accurate prediction of imagery motion from 2–2.5 s of session duration.

With respect to the related research, we used the brain waves α, β, δ, γ, and θ through TP9, TP10, AF7, and AF8 channels as in previous results [13], due to the accurate classification achieved. For that reason, we discarded raw data as in [25] or only the gamma waves of AF7 and AF8 channels used in [26]. We also prevented overfitting using dropout layers, regularizers, and early stopping. Moreover, we also tested different window and session sizes configurations to find the best setup. However, more importantly, our results go one step further, we achieved to reduce the session size from 20 s to 2 s getting an acceptable accuracy between 83% and 92% also with 2 s window size (1 single window). Thus, our DL model is available to predict the correct imagery motion in a time-response lower than the related work. Although 2 s of time-response is not a real-time (immediate) response, these results are promising overcoming all of the existing previous work (Table 1).

Furthermore, the participants reported a good usability of the system during the experiment. They felt very good wearing the headband, because it is comfortable, low-intrusive, and the data were collected transparently for them.

### 4.2. Experiment 2: Validating the Response Time Reduction

In order to validate the response time reduction achieved in the previous experiment, we obtained data from all of the participants recruited; 20 recordings per participant. The main idea behind this experiment is to validate the model for motor imagery classification for the 2 s session sizes, while a participant is imaging picking up bottles during a minute, changing the hand-side every 6 s. Since we recorded the data from the Mind Monitor app, we used labelling buttons for creating labels (marks) in the recordings. Thus, we know exactly when we asked the participant to change form right to left or vice versa. We selected 6 s to change sides, in order to cover the 2 s for classification and the possible delays. These delays involve asking the participant to change sides and to manually press labelling button in the app. This could imply a latency in these two actions (see Figure 12).

The number of trials consisted of 60 recordings in total (20 times 3), and every trial had 10 samples (5 for right; and 5 for left motor imagery). We created three different models, one per participant and reserved 20% of the recordings for testing (4 recordings per participant). Then, the resulting 16 recordings were split again into 80–20% for training and validation, respectively (12 for training; 4 for validation). In terms of samples, we used in total 120 samples for training the model, 40 samples for validation, and 40 samples for testing. We repeated this model creation process over 5 iterations in order to randomize the selection of recordings, and finally averaged the results of the 5 iterations.

We tested the DL pipeline for the 2 s time-response with different window sizes. The results are presented in Table 2. The best test accuracy averaged over the three participants was 83.8% using 0.5 s window size. One participant achieved the best test accuracy of 93% (the only male), followed by the other participants with 80.5% and 78%.

These results validate our previous set of experiments, confirming that in two seconds, we are able to classify the motor imagery with an accurate value. These results are not exactly the same as in the first set of experiments, partially due to the different participants. It is well-known that the brain activity is quite personal and can change over time [46]. Although we can have some general patterns, neither all the persons have the same sensibility in brain activity, nor the same concentration ability [46]. In any case, these results overcome the related works using the Muse headband (low-cost EEG device), reducing time-response and maintaining a decent accuracy. Furthermore, our accuracy results also overcome the related works which reduce the time-response in the range of 2–4 s (these studies used high-intrusive devices). The best work [36] reached 93.9% accuracy (but with 4 s time-response), followed by [39,40] with 90.5% accuracy (3 s time-response), and [36] with 70% accuracy (2 s time-response). The underlined differences on the motor imagery classification among the participants should be study further with the help of brain specialists, and study the innate skills on imaging the hand-movement of each participant.

## 5. Conclusions and Future Work

In this paper, we have explored what is the smallest response time that still obtains a competitive accuracy on the detection of motor imagery using a low-cost and low-intrusive EEG headband. We used a deep learning pipeline, based on 1D-CNN and LSTM layers, to explore the best reduction of the response time in motor imagery classification with a low-cost and low-intrusive BCI headband.

Our results showed that motor imagery classification for left and right hands, with low-cost and low-intrusive headband devices (with 4 electrodes), is feasible, accurate, and can reduce the time-response to 2 s. The proposed deep learning model architecture, based on 1D-CNN and LSTM layers, ensures an accuracy between 83% and 92% with unseen data for a time-response of 2 s or longer. These results overcome related works with intrusive and with non-intrusive EEG devices. High-intrusive EEG caps, with hundreds of electrodes, reach accuracies between 40% and 93.9% and provide a time-response of 4 s (two times slower than our architecture). Low-intrusive devices in the literature use session sizes greater than 10 s.

We have found that low-intrusive devices (headbands) can compete with high-intrusive devices in some tasks; in particular, in motor imagery for right and left hand. Although the electrodes of the headbands do not cover the primary cortex area (C3 and C4 electrodes), they cover the frontal lobe of the brain, which is related with movement control functions of the brain. Hence, the headbands could still detect movement (motor) imagery.

This proposal has the potential of increasing people’s independence, allowing them to interact with computer systems and applications without using their bodies, but just thinking or imagining the movement.

In the future, we would like to increase the sample size to enhance the accuracy, and also to apply our algorithms to other participants comparing not only the participants, but also to further study the personal responses and how they affect the personalities in the classification. Additionally, we want to compare the effect of different percentages of overlapping sliding windows. We will also explore the possibility to further reduce the response time. This is not an irrelevant task, as in real life we have to offer an immediate response, and 2 s, which is reported as the best accuracy in our experiments, could slow down the user experience. Furthermore, although the headband used in this work is low-intrusive compared to similar EEG devices, it is still awkward to wear in outdoors activities. In the future, probably other non-intrusive devices will appear, which will be suitable for outdoors activities, and we would like to study them. These next-generation devices may also have more electrodes than the Muse and located on motor cortex (maintaining the low-intrusiveness); and could improve the accuracy and response time. Furthermore, the properties of the Muse headband such as low-cost and low-intrusive contribute to democratize the adoption of these BCI wearable technologies in the health domain. In this line, another of our objectives is the recognition of activities, mental state, and emotions of the users while they are performing daily life activities and the relationship with their health status. The headband will be part of our previous e-health system based on microservices and the cloud [42,47], which includes other wearable sensors and applications to monitor and intervene in the healthcare of the elderly. Thus, the integration of the proposal presented in this paper with our previously designed e-health system will be able to contribute to a practical solution for healthcare systems.

## Figures and Tables

**Table 1:** Review of previous works about motor imagery using machine learning.

Work	Method	Channels	Intrusive	Own Dataset	Subjects	Classes	Session Size	Validation Split	Accuracy
[27]	CNN+LSTM	EEG: 64	Yes	No	Cross-Subject: 108	Five	120 s	75–25%	98.3%
[28]	LSTM	EEG: 64	Yes	No	Intra-Subject: 109	Five	120 s	5 × 5-fold	97.8%
[25]	CNN+LSTM	Muse: 4	Low	Yes	Intra-Subject: 1	Four	30 s	90–10%	80.13%
[13]	CNN+LSTM	Muse: 4	Low	Yes	Cross-Subject: 4	Binary	20 s	90–10%	98.9%
[26]	SVM	Muse: 4	Low	Yes	Intra-Subject: 8	Binary	10 s	4-fold	95.1%
[32]	SVM	EEG: 2	Yes	No	Intra-Subject: 2	Binary	9 s	50–50%	82.14%
[33]	CNN	EEG: 28	Yes	Yes	Intra-Subject: 2	Binary	5 s	80–20%	86.41%
[36]	RLDA CNN	EEG: 22 EEG: 44	Yes Yes	No Yes	Intra-Subject: 9 Intra-Subject: 20	Four Four	4 s 4 s	ICV	73.7% 93.9%
[35]	LSTM	EEG: 6	Yes	No	Intra-Subject: 9	Binary	4 s	5 × 5-fold	79.6%
[37]	CNN	EEG: 3	Yes	No	Intra-Subject: 9	Binary	4 s	60%–40%	78.44%
[34]	CNN+SAE	EEG: 3	Yes	No	Intra-Subject: 9	Binary	4 s	10 × 10-fold	77.6%
[39,40]	LR	EEG: 128	Yes	Yes	Intra-Subject: 29	Three	3 s	50%–50%	90.5%
[38]	CNN	EEG: 22	Yes	No	Intra-Subject: 9 Cross-Subject: 9	Four	2 s	4-fold	~70% ~40%

SVM: support vector machine; RLDA: regularized linear discriminant analysis; ICV: inner cross validation.

**Table 2:** Validation of DL models for 2 s time-response.

WS	TrAcc1	VaAcc1	TeAcc1	TrAcc2	VaAcc2	TeAcc2	TrAcc3	VaAcc3	TeAcc3	TrAvg	VaAvg	TeAvg
2 s	0.773846	0.760000	0.700000	0.795385	0.746667	0.705000	0.829231	0.786667	0.820000	0.799487	0.764444	0.741667
1.5 s	0.747692	0.753333	0.720000	0.807692	0.806667	0.725000	0.847692	0.880000	0.850000	0.801025	0.813333	0.765000
1 s	0.912308	0.840000	0.795000	0.829231	0.826667	0.740000	0.949231	0.920000	0.905000	0.896923	0.862222	0.813333
0.5 s	0.920000	0.833333	0.805000	0.906154	0.860000	0.780000	0.940000	0.913334	**0.930000**	0.922051	0.868889	**0.838333**
0.25 s	0.896923	0.873333	0.765000	0.832308	0.840000	0.745000	0.916923	0.873333	0.890000	0.882051	0.862222	0.800000
0.1 s	0.841538	0.833333	0.760000	0.803077	0.773333	0.700000	0.840000	0.853333	0.855000	0.828205	0.820000	0.771667
0.05 s	0.835384	0.866667	0.730000	0.692308	0.706667	0.720000	0.818462	0.853333	0.845000	0.782051	0.808889	0.765000

Averaged all accuracies from 5 iterations. Best test accuracies in bold letters. Best individual test-accuracy underlined. WS: window size; TrAccX: train accuracy of participant X; VaAccX: validation accuracy of participant X; TeAccX: test accuracy of participant X; TrAvg: train accuracy average; VaAvg: validation accuracy average; TeAvg: test accuracy average.

## Abbreviation

AFX	position of an electrode in BCI, with number X
BCI	brain computer interfaces
CNN	convolutional neural network
DL	deep learning
EEG	electroencephalography
Fpz	reference electrode in BCI
LR	logistic regression
LSTM	long short-term memory
ML	machine learning
NN	neural network
RNN	recurrent neural network
SDK	software development kits
SVM	support vector machines
TPX	position of an electrode in BCI, with number X
